# Sex disparities in mortality among patients with kidney failure receiving dialysis

**DOI:** 10.1038/s41598-022-16163-w

**Published:** 2022-11-03

**Authors:** Hee-Yeon Jung, Yena Jeon, Yon Su Kim, Shin-Wook Kang, Chul Woo Yang, Nam-Ho Kim, Hee-Won Noh, Soo-Jee Jeon, Jeong-Hoon Lim, Ji-Young Choi, Jang-Hee Cho, Sun-Hee Park, Chan-Duck Kim, Yong-Lim Kim

**Affiliations:** 1 Division of Nephrology, Department of Internal Medicine, School of Medicine, Kyungpook National University, Kyungpook National University Hospital, 130 Dongdeok-ro, Jung-gu, Daegu, 41944 South Korea; 2 Department of Statistics, Kyungpook National University, Daegu, South Korea; 3 Department of Internal Medicine, Seoul National University College of Medicine, Seoul, South Korea; 4 Department of Internal Medicine, Yonsei University College of Medicine, Seoul, South Korea; 5 Department of Internal Medicine, College of Medicine, The Catholic University of Korea, Seoul, South Korea; 6 Department of Internal Medicine, Chonnam National University Medical School, Gwangju, South Korea

**Keywords:** Diseases, Nephrology

## Abstract

Females are known to have a better survival rate than males in the general population, but previous studies have shown that this superior survival is diminished in patients on dialysis. This study aimed to investigate the risk of mortality in relation to sex among Korean patients undergoing hemodialysis (HD) or peritoneal dialysis (PD). A total of 4994 patients with kidney failure who were receiving dialysis were included for a prospective nationwide cohort study. Cox multivariate proportional hazard models were used to determine the association between sex and the risk of cause-specific mortality according to dialysis modality. During a median follow-up of 5.8 years, the death rate per 100 person-years was 6.4 and 8.3 in females and males, respectively. The female-to-male mortality rate in patients on dialysis was 0.77, compared to 0.85 in the general population. In adjusted analyses, the risk of all-cause mortality was significantly lower for females than males in the entire population (hazard ratio [HR] 0.79, 95% confidence interval [CI] 0.71–0.87, P < 0.001). No significant differences in the risk of cardiovascular and infection-related deaths were observed according to sex. The risk of mortality due to sudden death, cancer, other, or unknown causes was significantly lower for females than males in the entire population (HR 0.66, 95% CI 0.56–0.78, P < 0.001), in patients on HD (HR 0.75, 95% CI 0.62–0.90, P = 0.003), and in patients on PD (HR 0.49, 95% CI 0.34–0.70, P < 0.001). The survival advantage of females in the general population was maintained in Korean dialysis patients, which was attributed to a lower risk of noncardiovascular and noninfectious death.

**Trial registration**: ClinicalTrials.gov Identifier: NCT00931970.

## Introduction

Females have a higher survival rate than males in the general population^1^, which may be related to a lower prevalence of cardiovascular risk factors and cardiovascular disease in females^2,3^. As differences in male and female physiology have been recognized, sex-specific distinctions have been widely reported for many diseases, including type 2 diabetes mellitus, cardiovascular disease, depression, acute kidney injury, chronic kidney disease, and kidney failure treated by dialysis^4–15^.

Previous analyses^13–16^, including studies from the Dialysis Outcomes and Practice Patterns Study (DOPPS) and the Austrian Dialysis Registry, have shown that the superior survival rate of females compared to males in the general population is not maintained in the dialysis population. This cancellation of the survival advantage for females was largely independent of differences in comorbidities or smoking status, and the reasons for this finding have not been fully understood. Considering that ethnicity, practice patterns, and accessibility to medical care could affect dialysis outcomes, it is important to investigate sex disparities in the survival of Asian patients on dialysis across countries.

This nationwide prospective cohort study in Korea aimed to investigate whether the survival benefit of females compared to males in the general population persist in the kidney failure population and to determine the association between sex and the risk of cause-specific mortality.

## Results

### Patient characteristics

**Figure 1:**
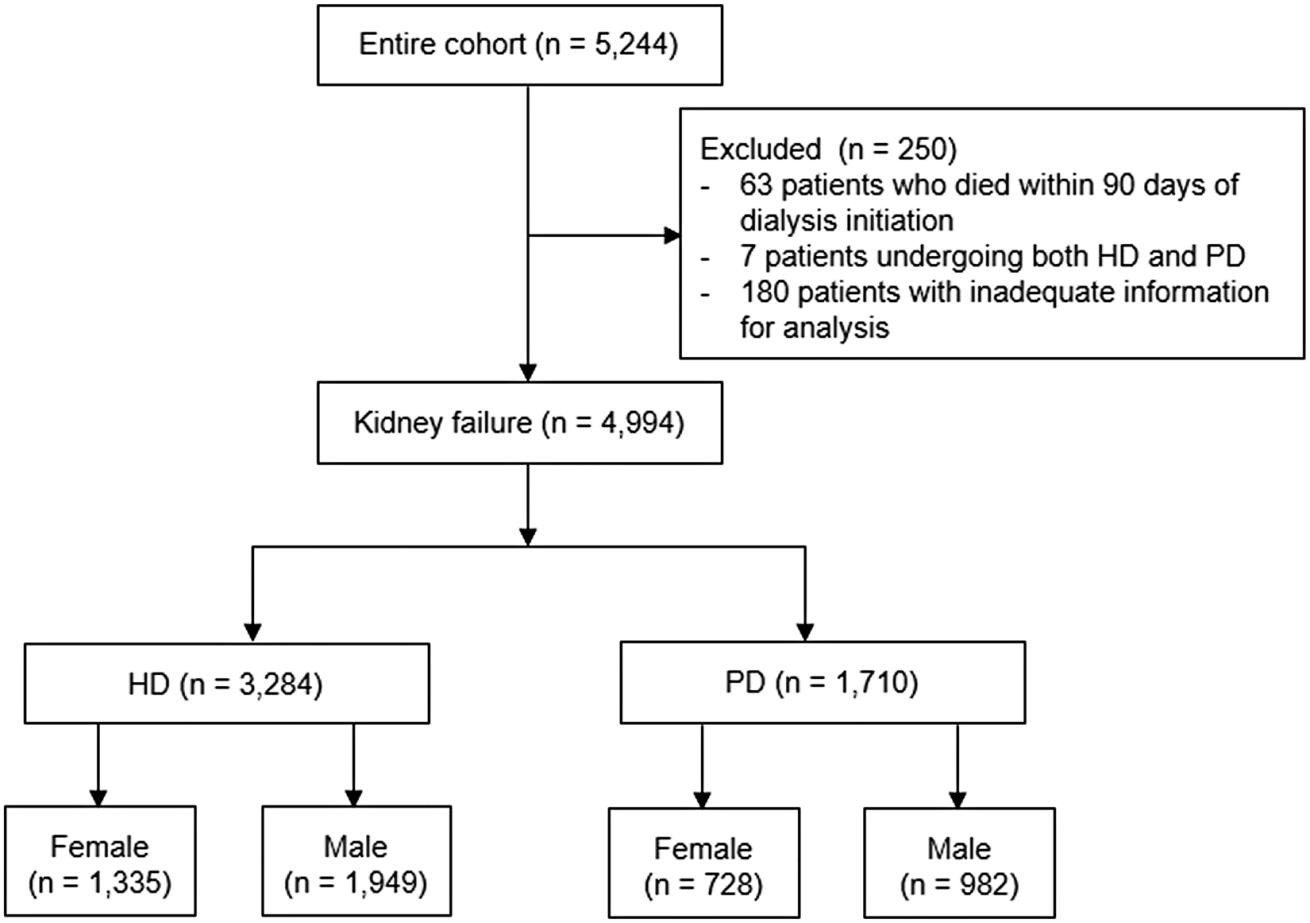
Flow chart of participant inclusion. Among a total of 5244 incident and prevalent patients receiving dialysis, 63 patients who died within 90 days of dialysis initiation, 7 patients undergoing both hemodialysis (HD) and peritoneal dialysis (PD), and 180 patients with inadequate information for analysis were excluded. Thus, a total of 3284 patients on HD and 1710 patients on PD were included in this study.

Figure 1 shows the patient inclusion process. Among a total of 5244 incident and prevalent patients receiving dialysis, 63 patients who died within 90 days of dialysis initiation, 7 patients undergoing both HD and PD, and 180 patients with inadequate information for analysis were excluded from this study. Thus, a total of 3284 patients on HD and 1710 patients on PD were included in this study.

Baseline characteristics are provided in Table 1. In the entire study population, females had lower body mass index (BMI), a lower proportion of diabetes as the primary kidney disease, and fewer comorbidities than males. The proportion of incident patients was significantly higher among male patients than female patients and, accordingly, the dialysis vintage was significantly shorter in male patients than female patients. The rate of transition to transplantation did not differ between the two groups.

**Table 1:** Baseline characteristics of the study populations.

	All			HD			PD		
	Female (n = 2063)	Male (n = 2931)	P-value	Female (n = 1335)	Male (n = 1949)	P-value	Female (n = 728)	Male (n = 982)	P-value
Age at initiation of dialysis (years)	55.7 ± 13.6	56.5 ± 13.5	0.050	58.3 ± 13.6	58.1 ± 13.7	0.742	51.1 ± 12.4	53.3 ± 12.6	< 0.001
**Population type**									
Incident	1125 (54.5)	1773 (60.5)	< 0.001	784 (58.7)	1261 (64.7)	< 0.001	341 (46.8)	512 (52.1)	0.030
Prevalent	938 (45.5)	1158 (39.5)		551 (41.3)	688 (35.0)		387 (53.2)	470 (47.9)	
Dialysis vintage (years)	8.0 (6.1–10.6)	7.7 (5.8–9.8)	< 0.001	7.9 (6.1–10.5)	7.5 (5.7–9.6)	< 0.001	8.3 (6.2–10.8)	8.0 (6.0–10.1)	0.024
Length of follow-up (years)	5.6 (3.4–7.2)	5.0 (2.9–6.8)	< 0.001	5.6 (3.4–7.2)	5.1 (2.8–6.8)	< 0.001	5.6 (3.3–7.5)	4.8 (2.9–6.8)	< 0.001
Transition to kidney transplantation	435 (21.1)	614 (21.0)	0.907	216 (16.2)	344 (17.7)	0.271	219 (30.1)	270 (27.5)	0.242
BMI (kg/m^2^)	22.7 ± 3.6	23.0 ± 3.1	0.004	22.6 ± 3.7	22.7 ± 3.2	0.164	23.0 ± 3.5	23.5 ± 2.8	0.001
**Primary kidney disease**									
Diabetes	951 (46.1)	1582 (54.0)	< 0.001	695 (52.1)	1096 (56.2)	0.131	256 (35.2)	486 (49.5)	< 0.001
Hypertension	385 (18.7)	467 (15.9)		208 (15.6)	280 (14.4)		17 (24.3)	187 (19.0)	
CGN	306 (14.8)	355 (12.1)		161 (12.1)	217 (11.1)		145 (19.9)	138 (14.1)	
Other	421 (20.4)	527 (18.0)		271 (20.3)	356 (18.3)		150 (50.6)	171 (17.4)	
**Comorbidity**									
Coronary artery disease	198 (9.7)	412 (14.1)	< 0.001	144 (10.9)	305 (15.7)	< 0.001	54 (7.4)	107 (10.9)	0.014
Cerebrovascular disease	141 (6.9)	251 (8.6)	0.027	108 (8.1)	184 (9.5)	0.192	33 (4.6)	67 (6.8)	0.047
Congestive heart failure	194 (9.5)	263 (9.0)	0.587	137 (10.3)	185 (9.5)	0.441	57 (7.8)	78 (8.0)	0.922
Arrhythmia	51 (2.5)	82 (2.8)	0.488	39 (2.9)	63 (3.2)	0.627	12 (1.7)	19 (1.9)	0.657
Peripheral vascular disease	90 (4.4)	194 (6.7)	0.001	59 (4.5)	144 (7.4)	< 0.001	31 (4.3)	50 (5.1)	0.422
Hypertension	385 (18.7)	467 (15.9)	0.012	208 (15.6)	280 (14.4)	0.337	177 (24.3)	187 (19.0)	0.009
Chronic lung disease	94 (4.6)	198 (6.8)	0.001	68 (5.1)	148 (7.6)	0.005	26 (3.6)	50 (5.1)	0.132
Moderate to severe chronic liver disease	38 (1.9)	119 (4.1)	< 0.001	24 (1.8)	90 (4.6)	< 0.001	14 (1.9)	29 (3.0)	0.179
Malignancy	119 (5.8)	141 (4.8)	0.131	88 (6.7)	123 (6.4)	0.728	31 (4.3)	18 (1.8)	0.003
**Laboratory data**									
Hemoglobin (g/dL)	9.9 ± 1.6	9.9 ± 1.7	0.658	9.8 ± 1.6	9.7 ± 1.7	0.232	10.0 ± 1.6	10.2 ± 1.6	0.007
Albumin (g/dL)	3.6 ± 0.5	3.6 ± 0.6	0.218	3.6 ± 0.5	3.6 ± 0.6	0.242	3.6 ± 0.5	3.6 ± 0.5	0.560
Calcium (mg/dL)	8.5 ± 1.1	8.3 ± 1.0	< 0.001	8.4 ± 1.1	8.2 ± 1.1	< 0.001	8.6 ± 1.0	8.3 ± 1.0	< 0.001
Phosphorus (mg/dL)	5.1 ± 1.7	5.3 ± 1.8	< 0.001	5.1 ± 1.7	5.3 ± 1.8	0.003	5.1 ± 1.6	5.3 ± 1.7	0.003
**SGA scores**									
Score 6–7 (well nourished)	1396 (74.2)	2058 (76.7)	0.051	907 (71.8)	1393 (75.7)	0.015	489 (79.3)	665 (79.1)	0.933
Score 1–5 (mildly to severely malnourished)	485 (25.8)	624 (23.3)		357 (28.2)	448 (24.3)		128 (20.8)	176 (20.9)	

Values are given as mean ± standard deviation, median (range), or n (%).

*BMI* body mass index, *CGN* chronic glomerulonephritis, *HD* hemodialysis, *PD* peritoneal dialysis, *SGA* subjective global assessment.

### Causes of death

**Figure 2:**
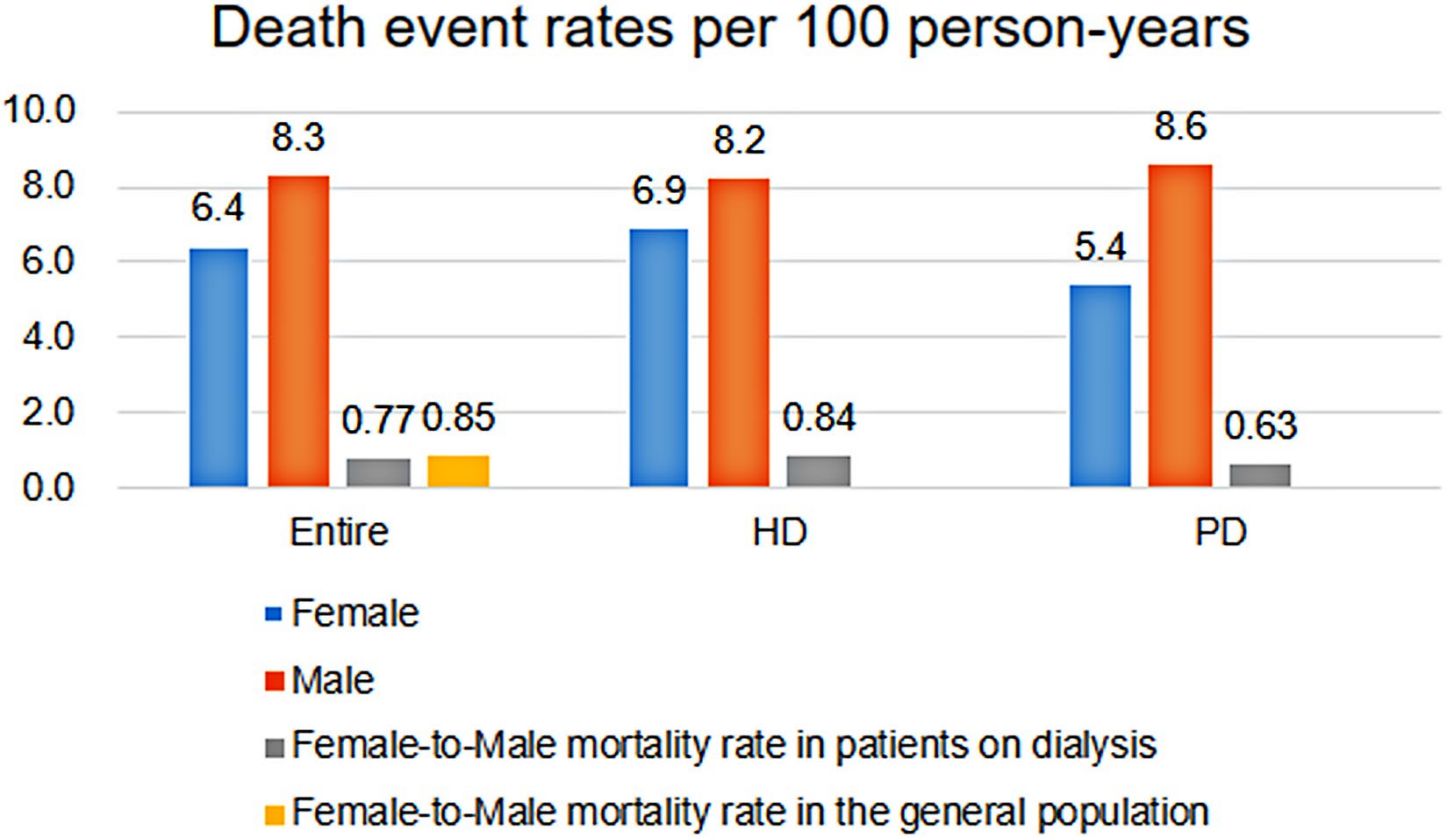
Death event rates per 100 person-years in male and female patients according to dialysis modality. The rate was 6.4 in females and 8.3 in males in the entire population, 6.9 in females and 8.2 in males in the hemodialysis (HD) population, and 5.4 in females and 8.6 in males in the peritoneal dialysis (PD) population.

During the median follow-up of 5.8 (IQR 3.6–7.4) years, 678 (32.9%) and 1163 deaths (39.7%) occurred in females and males, respectively. The rate of death per 100 person-years was 6.4 in females and 8.3 in males in the entire population. The female-to-male mortality rate in patients on dialysis was 0.77, which was comparable to the 0.85 female-to-male mortality rate in the general population^17^. The rate of death per 100 person-years in the HD population was 6.9 in females and 8.2 in males. In the PD population, the rates were 5.4 in females and 8.6 in males (Fig. 2). Cardiovascular death was the most common cause of death in both dialysis modalities (Table 2).

**Table 2:** Causes of death.

Cause of death	All			HD			PD		
	Female (n = 2063)	Male (n = 2931)	P-value	Female (n = 1335)	Male (n = 1949)	P-value	Female (n = 728)	Male (n = 982)	P-value
Cardiovascular death	285 (42.0)	451 (38.8)	0.209	196 (41.4)	312 (40.8)	0.411	89 (43.4)	139 (34.8)	0.032
Infection-related death	146 (21.5)	234 (20.1)		88 (18.6)	142 (18.6)		58 (28.3)	92 (23.1)	
Cancer	43 (6.3)	109 (9.4)		36 (7.6)	86 (11.3)		7 (3.4)	23 (5.8)	
Sudden death	86 (12.7)	163 (14.0)		68 (14.4)	100 (13.1)		18 (8.8)	63 (15.8)	
Other	75 (11.1)	125 (10.8)		60 (12.7)	84 (11.0)		15 (7.3)	41 (10.3)	
Unknown	43 (6.3)	81 (7.0)		25 (5.3)	40 (5.2)		18 (8.8)	41 (10.3)	
Total	678 (100)	1163 (100)		473 (100)	764 (100)		205 (100)	399 (100)	

Values are given as n (%).

*HD* hemodialysis, *PD* peritoneal dialysis.

### Risk of mortality by sex

**Figure 3:**
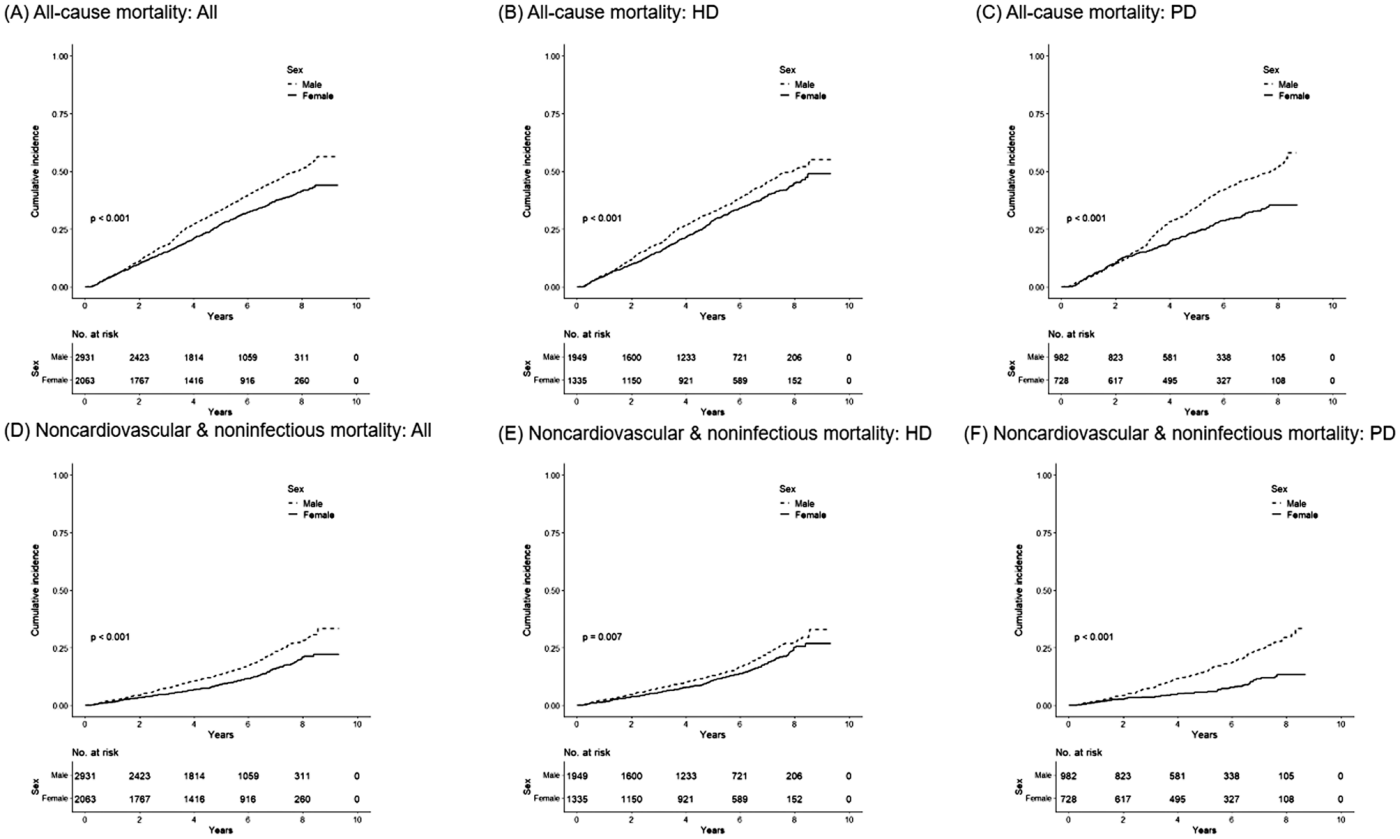
Cumulative incidence curve for deaths in male and female patients according to dialysis modality. (**A**–**C**) Male patients had significantly more all-cause death events and (**D**–**F**) noncardiovascular and noninfectious death events than female patients in the entire population, hemodialysis (HD) population, and peritoneal dialysis (PD) population.

In the cumulative incidence curve, males had significantly more all-cause death events and noncardiovascular and noninfectious death events than females in the entire population, the HD population, and the PD population (Fig. 3).

Cox multivariate proportional hazard models (Table 3) demonstrated that females had a significantly lower risk of all-cause death than males in the entire population (hazard ratio [HR] 0.79, 95% confidence interval [CI] 0.71–0.87, P < 0.001). The risk of mortality due to sudden death, cancer, other, or unknown causes was significantly lower for females than males in the entire population (HR 0.66, 95% CI 0.56–0.78, P < 0.001), in patients on HD (HR 0.75, 95% CI 0.62–0.90, P = 0.003), and in patients on PD (HR 0.49, 95% CI 0.34–0.70, P < 0.001) (Table 4). Multivariable regression analysis of the association between sex and mortality according to the type of population (incident or prevalent) showed consistent results (Supplementary Table S1). No significant differences in the risk of cardiovascular and infection-related deaths were observed according to sex.

**Table 3:** Multivariable regression analysis of the association of sex (female versus male) with all-cause mortality.

All-cause mortality	All		HD		PD	
	aHR_F:M_ (95% CI)	P-value	aHR_F:M_ (95% CI)	P-value	aHR_F:M_ (95% CI)	P-value
Model 1	0.78 (0.71–0.85)	< 0.001	0.81 (0.72–0.91)	< 0.001	0.74 (0.62–0.88)	< 0.001
Model 2	0.83 (0.76–0.92)	< 0.001	0.86 (0.76–0.96)	0.010	0.86 (0.72–1.02)	0.079
Model 3	0.81 (0.74–0.89)	< 0.001	0.84 (0.75–0.94)	0.003	0.82 (0.68–0.97)	0.024
Model 4	0.79 (0.71–0.87)	< 0.001	0.82 (0.73–0.92)	0.001	0.77 (0.65–0.92)	0.005

*CI* confidence interval, *F* female, *aHR* adjusted hazard ratio, *HD* hemodialysis, *M* male, *PD* peritoneal dialysis.

Model 1: Adjusted for age at the time of dialysis.

Model 2: Model 1 + adjusted for body mass index, diabetes, cardiovascular comorbidities (coronary artery disease, cerebrovascular disease, congestive heart failure, arrhythmia, peripheral vascular disease, hypertension), chronic lung disease, moderate to severe chronic liver disease, and malignancy.

Model 3: Model 2 + adjusted for albumin, hemoglobin, calcium, and phosphorus.

Model 4: Model 3 + adjusted for subjective global assessment scores and dialysis vintage.

**Table 4:** Multivariable regression analysis of the association of sex (female versus male) according to cause-specific mortality.

	All		HD		PD	
	aHR_F:M_ (95% CI)	P-value	aHR_F:M_ (95% CI)	P-value	aHR_F:M_ (95% CI)	P-value
Cardiovascular mortality	0.90 (0.77–1.05)	0.179	0.85 (0.70–1.02)	0.083	1.14 (0.84–1.56)	0.392
Infection-related mortality	0.92 (0.73–1.15)	0.450	0.89 (0.67–1.19)	0.428	1.05 (0.72–1.53)	0.807
Noncardiovascular and noninfectious mortality	0.66 (0.56–0.78)	< 0.001	0.75 (0.62–0.90)	0.003	0.49 (0.34–0.70)	< 0.001

*CI* confidence interval, *F* female, *aHR* adjusted hazard ratio, *HD* hemodialysis, *M* male, *PD* peritoneal dialysis.

Models were adjusted for age at the time of dialysis, body mass index, diabetes, cardiovascular comorbidities (coronary artery disease, cerebrovascular disease, congestive heart failure, arrhythmia, peripheral vascular disease, hypertension), chronic lung disease, moderate to severe chronic liver disease, malignancy, albumin, hemoglobin, calcium, phosphorus, subjective global assessment scores, and dialysis vintage.

### Sex-specific factors for death and interaction analyses

Table 5 shows factors associated with all-cause mortality according to sex in the multivariate regression analysis. Older age at the time of dialysis initiation, the presence of diabetes, cardiovascular comorbidities, moderate to severe chronic liver disease, and lower level of serum albumin were independent risk factors for all-cause mortality in both females and males. Mild to severe malnutrition (adjusted HR 1.27, 95% CI 1.10–1.46, P = 0.001) was an independent risk factor for mortality in males, but not in females.

**Table 5:** Factors associated with all-cause mortality according to sex in the multivariable regression analysis.

	Female		Male	
	aHR (95% CI)	P-value	aHR (95% CI)	P-value
Age at the time of dialysis	1.05 (1.05–1.06)	< 0.001	1.05 (1.05–1.06)	< 0.001
BMI	0.98 (0.96–1.01)	0.218	0.98 (0.96–1.00)	0.064
Diabetes	1.90 (1.60–2.26)	< 0.001	1.76 (1.54–2.01)	< 0.001
Cardiovascular comorbidities	1.43 (1.22–1.68)	< 0.001	1.35 (1.19–1.53)	< 0.001
Moderate to severe chronic liver disease	1.91 (1.17–3.11)	0.010	1.35 (1.03–1.77)	0.030
Albumin	0.62 (0.52–0.74)	< 0.001	0.66 (0.58–0.75)	< 0.001
SGA score 1–5 vs. 6–7	1.09 (0.91–1.30)	0.365	1.27 (1.10–1.46)	0.001

*BMI* body mass index, *CI* confidence interval, *aHR* adjusted hazard ratio, *SGA* subjective global assessment.

Models were adjusted for age at the time of dialysis, BMI, diabetes, cardiovascular comorbidities, chronic lung disease, moderate to severe chronic liver disease, malignancy, albumin, hemoglobin, calcium, phosphorus, SGA scores, and dialysis vintage.

**Figure 4:**
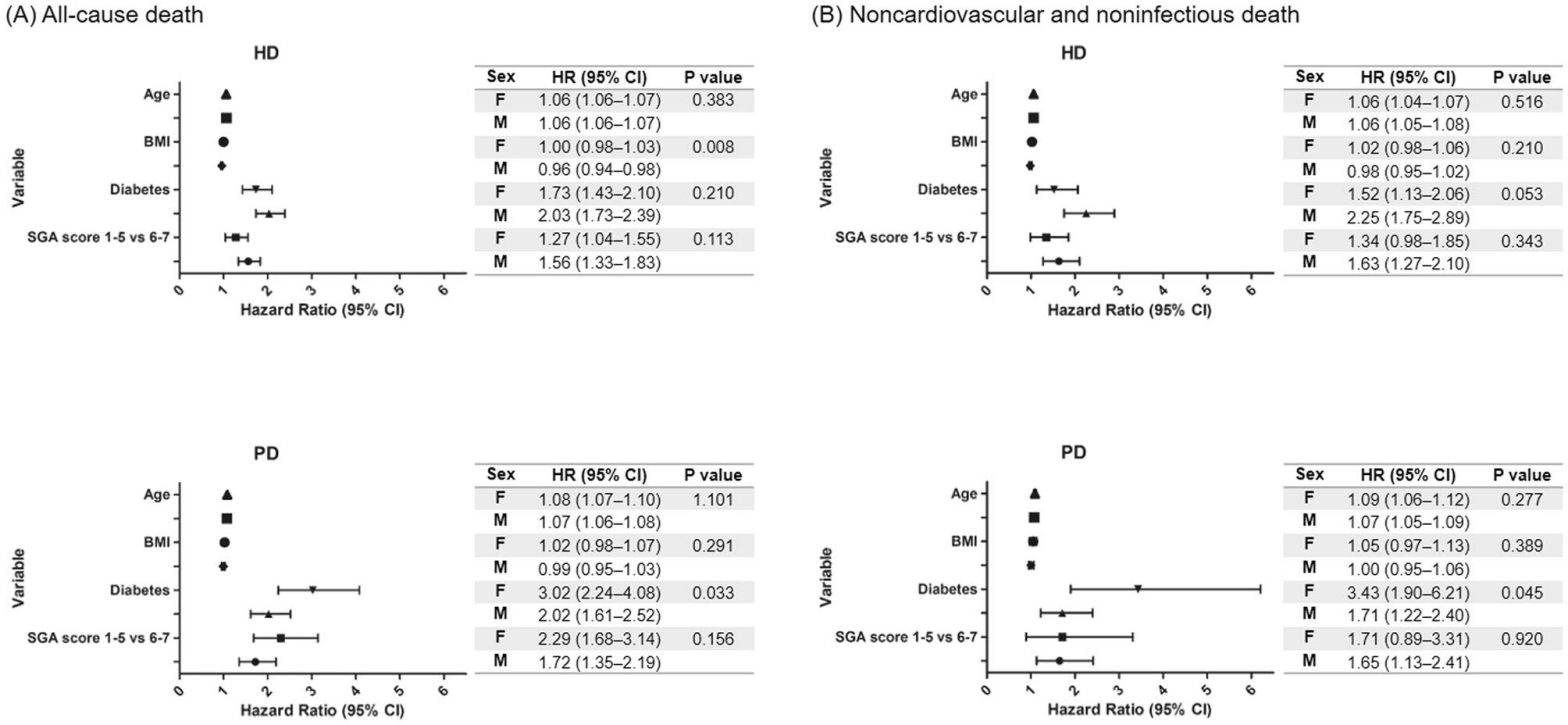
(**A**) Analysis of sex interaction in the associations between patient characteristics and all-cause or (**B**) noncardiovascular and noninfectious death. The risk of all-cause mortality associated with diabetes was higher in female patients on peritoneal dialysis (PD) than male patients on PD (interaction P = 0.033). The risk of noncardiovascular and noninfectious death associated with diabetes was higher in female patients on PD than in male patients on PD (interaction P = 0.045).

Interaction analyses (Fig. 4) showed that the risk of all-cause death (interaction P = 0.033) and noncardiovascular and noninfectious death (interaction P = 0.045) associated with diabetes was higher in female patients on PD than in male patients on PD. In addition, the risk of all-cause death associated with BMI was lower in male patients on HD than in female patients on HD (interaction P = 0.008).

## Discussion

In this nationwide prospective cohort study, females with kidney failure receiving HD or PD exhibited a lower risk of death than males during a median follow-up of 6.3 years after extensive adjustment for demographic factors, cardiovascular and noncardiovascular comorbidities, laboratory findings, and nutritional status. The survival benefit of females on dialysis was mainly attributed to a lower risk of noncardiovascular and noninfectious death. Furthermore, factors associated with all-cause death differed in males and females with kidney failure. Mild to severe malnutrition and lower BMI were independent risk factors for mortality in males but not in females.

In contrast to the results from this Korean nationwide cohort study, previous studies^13–16^, including international dialysis cohort studies, have reported that the survival advantage of females compared to males in the general population is diminished in the HD population. Though the mortality of males surpassed that of females in the general population in all DOPPS countries, with male-to-female mortality rate ratios > 2 in certain countries, mortality rates were very similar for males and females in the HD population, with male-to-female mortality rate ratios close to 1 in all DOPPS countries except Japan^14^. The varying results may be explained by ethnicity, different practice patterns, differences in access to health care due to different insurance systems between countries, and various unadjusted biases. Previous studies explained that similar survival rates by sex in HD patients may be due to the mortality risk associated with diabetes, coronary artery disease, and cardiovascular disease^14^, or that non-cardiovascular disease^18^ was higher among adult females than male HD patients. Moreover, higher BMI, which is well known to be associated with better survival in HD patients^19,20^, was reported to be slightly less protective among females than males^14^. Cardiovascular mortality between male and female dialysis patients in our study was similar to previous studies. However, the risk of noncardiovascular and noninfectious death was significantly lower in females than males on both HD and PD. Although the exact mechanism of this result may be uncertain, it provides important information that care for cardiovascular infectious complications, as well as other medical conditions, including respiratory, liver, gastro-intestinal, and endocrine or hematological diseases may be more necessary, especially in male dialysis patients.

This current study found that factors associated with death differed between males and females with kidney failure. Mild to severe malnutrition was an independent risk factors for mortality in male, but not female, patients on dialysis. In this study, analysis of the sex interaction showed that the risk of all-cause death associated with BMI was lower in male patients on HD than in female patients on HD. Previous studies from the present Korean kidney failure cohort have reported that BMI or subjective global assessment (SGA) could be useful for predicting mortality in male patients on HD but not in female patients on HD^21,22^. A greater protective effect of higher BMI on survival in male patients on dialysis than female patients on dialysis could be explained by higher skeletal muscle mass and reduced fat mass associated with sex hormones in males^23^ and a sex-specific association of skeletal muscles mass and arterial stiffness in dialysis patients^24^. Although the exact mechanism of death being less influenced by nutritional status in female patients on dialysis than male patients on dialysis is unclear, the possibility of other complementary factors associated with reduced vulnerability to malnutrition in females compared to males cannot be completely excluded.

In previous studies including the general population, the negative effect of diabetes on cardiovascular death was greater in females than in males^25,26^. In this study, analysis of the sex interaction showed that the risk of all-cause and noncardiovascular and noninfectious death associated with diabetes was significantly higher in females on PD than in males on PD. Similarly, the European Renal Association European Dialysis and Transplant Association Registry demonstrated that females with diabetes on PD have a higher mortality risk than males on PD^27^. Considering that the impact of diabetes on death differed according to dialysis modality and sex, individualized advice according to sex may be required in determining the dialysis modality, and tailored care according to sex may also be required in the management of dialysis patients.

This present study has some limitations. First, although we tried to adjust for significant confounding factors, unadjusted variables may have persisted. Second, misclassification of the cause of death cannot be completely excluded because the cause of death coding did not undergo an external audit. Third, the results from the present study showed only a significant association between mortality and sex in dialysis patients, not causation. Fourth, as the baseline information was obtained at the time of enrollment and some information, such as BMI, comorbidities, laboratory data, and SGA scores, can change over time, the information at the time of dialysis initiation may be reflected in incident patients, but not prevalent patients. Finally, sex was determined based on medical records and no information was available on transgender or nonbinary patients. Nevertheless, this study has several strengths. First, though previous international cohort studies have reported sex-specific differences in HD patients, this study provided results from both HD and PD patients, which can have different clinical outcomes. Second, this study is the first prospective cohort including an Asian population with relatively long follow-up period.

In conclusion, female Korean incident and prevalent dialysis patients on HD and PD had a survival benefit compared to male patients during a median follow-up period of 6.3 years, mainly due to a lower risk of noncardiovascular and noninfectious death. This suggests that monitoring and management for cardiovascular or infectious complications, as well as other causes of death from cancer, pulmonary, hepatic, gastro-intestinal disease, endocrine or hematological disease, and suicide, may be necessary, especially in male dialysis patients.

## Methods

### Study population

A nationwide prospective observational cohort study was conducted in Korean patients with kidney failure (NCT00931970). Patients who were at least 19 years old and who had initiated maintenance dialysis due to kidney failure within 3 months or received dialysis due to kidney failure for more than 3 months were eligible for the study. Patients scheduled for a kidney transplantation within 3 months were excluded from the study.

### Follow-up and outcomes

Incident or prevalent dialysis patients were enrolled from September 2008 to December 2013 and followed for the occurrence of death until December 2017. Patients were censored at the time of kidney transplantation. Cause of death was classified as all-cause, cardiovascular, or infection-related mortality, sudden death, cancer, other, or unknown. Cardiovascular death was defined as death from myocardial infarction, heart failure, arrhythmia, or stroke. Other causes of death included chronic obstructive lung disease, liver disease, gastro-intestinal disease, endocrine or hematological disease, and suicide.

### Other variables

Baseline information at the time of enrollment included age, sex, dialysis modality, dialysis vintage, BMI, SGA scores, comorbidities, and laboratory data. Comorbid conditions included a history of diabetes, coronary artery disease, cerebrovascular disease, congestive heart failure, peripheral vascular disease, arrhythmia, hypertension, chronic lung disease, moderate-to-severe chronic liver disease, and malignancy. Laboratory data included hemoglobin, albumin, calcium, and phosphorus. Dialysis modality was defined as the modality 90 days after the first dialysis.

### Statistical analysis

Data were expressed as mean ± standard deviation or median and interquartile range (IQR). Differences between groups were tested by independent sample t-tests and chi-squared tests as appropriate.

The Cox proportional hazard model adjusted for confounding factors was used to analyze the association between sex and mortality. Adjusted confounding factors included age, BMI, diabetes as the primary kidney disease, coronary artery disease, cerebrovascular disease, congestive heart failure, peripheral vascular disease, arrhythmia, hypertension, chronic lung disease, moderate-to-severe chronic liver disease, malignancy, hemoglobin, albumin, calcium, phosphorus, and SGA scores. Multivariable regression analysis was used to investigate the factors associated with all-cause mortality according to sex. The sex interaction in the association between patient characteristics and mortality was also analyzed.

Statistical analyses were performed using the SAS system for Windows, version 9.4 (SAS Institute Inc., Cary, NC) and R (R Foundation for Statistical Computing, Vienna, Austria; www.r-project.org). Significance was set at P < 0.05.

### Ethics approval and consent to participate

All patients provided written informed consent before inclusion, and the Institutional Review Board of each center approved the study protocol (in alphabetical order: The Catholic University of Korea, Bucheon St. Mary’s Hospital; The Catholic University of Korea, Incheon St. Mary’s Hospital; The Catholic University of Korea, Seoul St. Mary’s Hospital; The Catholic University of Korea, St. Mary’s Hospital; The Catholic University of Korea, St. Vincent’s Hospital; The Catholic University of Korea, Uijeongbu St. Mary’s Hospital; Cheju Halla General Hospital; Chonbuk National University Hospital; Chonnam National University Hospital; Chung-Ang University Medical Center; Chungbuk National University Hospital; Chungnam National University Hospital; Dong-A University Medical Center; Ehwa Womans University Medical Center; Fatima Hospital, Daegu; Gachon University Gil Medical Center; Inje University Pusan Paik Hospital; Kyungpook National University Hospital; Kwandong University College of Medicine, Myongji Hospital; National Health Insurance Corporation Ilsan Hospital; National Medical Center; Pusan National University Hospital; Samsung Medical Center, Seoul; Seoul Metropolitan Governmalet, Seoul National University, Boramae Medical Center; Seoul National University Hospital; Seoul National University, Bundang Hospital; Yeungnam University Medical Center; Yonsei University, Severance Hospital; Yonsei University, Gangnam Severance Hospital; Ulsan University Hospital; Wonju Christian Hospital). All clinical investigations were conducted in accordance with the guidelines of the Declaration of Helsinki and the Good Clinical Practice guidelines.

## Supplementary Information

Supplementary Information.

## Data Availability

Data supporting the findings of the current study are available from the corresponding author upon reasonable request.
