# Supplementary material for: Sex disparities in mortality among patients with kidney failure receiving dialysis
Source: Sci Rep. 2022 Nov 3;12:18555. doi: 10.1038/s41598-022-16163-w (PMC9633833; doi:10.1038/s41598-022-16163-w)
Supplement: Supplementary file 1 — Supplementary Information. [file 41598_2022_16163_MOESM1_ESM.docx]

Supplementary Table S1. Multivariable regression analysis of the association between sex and mortality according to the type of population

|  | All |  | HD |  | PD |  |
| --- | --- | --- | --- | --- | --- | --- |
| All-cause mortality | aHR_F:M_ (95% CI) | P-value | aHR_F:M_ (95% CI) | P-value | aHR_F:M_ (95% CI) | P-value |
| Incident | 0.78 (0.67–0.90) | <0.001 | 0.77 (0.65–0.91) | 0.002 | 0.86 (0.62–1.19) | 0.361 |
| Prevalent | 0.81 (0.70–0.93) | 0.003 | 0.83 (0.70–1.00) | 0.046 | 0.82 (0.64–1.05) | 0.114 |
| Noncardiovascular and noninfectious mortality |  |  |  |  |  |  |
| Incident | 0.68 (0.54–0.86) | 0.001 | 0.75 (0.58–0.96) | 0.025 | 0.50 (0.29–0.88) | 0.016 |
| Prevalent | 0.62 (0.49–0.80) | <0.001 | 0.71 (0.53–0.95) | 0.022 | 0.52 (0.33–0.82) | 0.005 |

Abbreviations: CI, confidence interval; F, female; aHR, adjusted hazard ratio; HD, hemodialysis; M, male; PD, peritoneal dialysis

Adjusted for age at the time of dialysis, body mass index, diabetes, cardiovascular comorbidities (coronary artery disease, cerebrovascular disease, congestive heart failure, arrhythmia, peripheral vascular disease, hypertension), chronic lung disease, moderate to severe chronic liver disease, malignancy, albumin, hemoglobin, calcium, phosphorus, subjective global assessment scores, and dialysis vintage.
